# Near-infrared spectroscopy (NIRS)-based eyes-closed brain-computer interface (BCI) using prefrontal cortex activation due to mental arithmetic

**DOI:** 10.1038/srep36203

**Published:** 2016-11-08

**Authors:** Jaeyoung Shin, Klaus-R Müller, Han-Jeong Hwang

**Affiliations:** 1 Machine Learning Group, Berlin Institute of Technology (TU Berlin), Marchstr. 23, 10587 Berlin, Germany; 2 Department of Brain and Cognitive Engineering, Korea University, 136-713 Seoul, Korea; 3 Department of Medical IT Convergence Engineering, Kumoh National Institute of Technology, 730-701 Gumi, Korea

## Abstract

We propose a near-infrared spectroscopy (NIRS)-based brain-computer interface (BCI) that can be operated in eyes-closed (EC) state. To evaluate the feasibility of NIRS-based EC BCIs, we compared the performance of an eye-open (EO) BCI paradigm and an EC BCI paradigm with respect to hemodynamic response and classification accuracy. To this end, subjects performed either mental arithmetic or imagined vocalization of the English alphabet as a baseline task with very low cognitive loading. The performances of two linear classifiers were compared; resulting in an advantage of shrinkage linear discriminant analysis (LDA). The classification accuracy of EC paradigm (75.6 ± 7.3%) was observed to be lower than that of EO paradigm (77.0 ± 9.2%), which was statistically insignificant (*p* = 0.5698). Subjects reported they felt it more comfortable (*p* = 0.057) and easier (*p* < 0.05) to perform the EC BCI tasks. The different task difficulty may become a cause of the slightly lower classification accuracy of EC data. From the analysis results, we could confirm the feasibility of NIRS-based EC BCIs, which can be a BCI option that may ultimately be of use for patients who cannot keep their eyes open consistently.

Near-infrared spectroscopy (NIRS) is an emerging neuroimaging technology which can monitor cortical activation using near-infrared light in the range of 600–900 nm. NIRS has many advantages: it is non-invasive, easy to use, has relatively low cost and is portable^1^. Many researchers have used NIRS to monitor cortical activity since JJ Jobsis first, in 1977, used NIRS to measure cerebral state changes when hyperventilating voluntarily^2^. Recently, NIRS has been also utilized for brain-computer interface (BCI) researches^3^,^4^ that aim at establishing a new communication modality for severely paralyzed patients using only their brain signals. Conventional NIRS-based BCIs have generally used left- and right-hand motor imagery tasks to modulate discriminable hemodynamic responses on the motor cortex^5^,^6^,^7^,^8^,^9^,^10^,^11^,^12^,^13^,^14^,^15^. However, since there are typically many hairs on the scalp above the motor cortex which interfere with near-infrared light, it is necessary to brush aside the hairs from the measuring location before the experiment.

To avoid this time-consuming preparation, measuring prefrontal cortex (PFC) activations may provide a better option because the forehead over PFC is a non-hair bearing area. More importantly, it has been well established that PFC plays an important role in processing cognitive tasks^16^. For example, Pfurtscheller *et al*.^17^ showed a concentration of oxy-hemoglobin (HbO) increase accompanied by a concentration of deoxy-hemoglobin (HbR) decrease over anterior PFC (APFC). So far, many NIRS-based BCI studies have showed promising results by using PFC activity^18^,^19^,^20^. Mental arithmetic (**MA**; e.g., successive subtraction of a small number from a large number) is known as one of the most robust tasks that reliably activate PFC areas^18^,^19^,^20^,^21^,^22^,^23^,^24^,^25^,^26^.

Traditionally, the eyes-closed (EC) state has been considered idle or resting state. Under this condition, the alpha rhythm (8–12 Hz) of the electroencephalogram (EEG) is strongly pronounced, especially around the occipital, parietal and posterior temporal regions^27^. It has been found that EEG and blood oxygenation level dependent (BOLD) signals are highly correlated under EC resting state^27^. Increased alpha rhythm power was associated with decreased BOLD signal in occipital, superior temporal, inferior frontal, cingulate cortices and with increased BOLD signal in the thalamus and insula^27^. This result indicates that the EC state does not significantly influence PFC activation. Hence we assume that a NIRS-based BCI paradigm mainly employing the PFC regions could be also applicable in EC state.

The development of EC BCI systems is fundamentally required for severely locked-in patients who are considered main users of BCI technology because they gradually lose their oculomotor functions in progressed states of the disease. Thus, they would have difficulties in using conventional BCI paradigms requiring normal or moderate visual functions for command selection and/or feedback. Two recent EEG-based BCI studies showed the feasibility of EC BCI paradigms^28^,^29^. One study used the steady-state visual evoked potential (SSVEP) paradigm^28^ and the other used the visual P300 paradigm^29^. Both studies showed acceptable classification results. Also, Gallegos-Ayala *et al*.^30^ recently reported the possibility of a NIRS-based EC BCI with a completely locked-in state (CLIS) patient but PFC was not used as the region of interest.

In this study, we investigated the possibility of developing an EC BCI based on self-modulated NIRS signals by performing MA. The performance of a traditional NIRS-based BCI with eyes-open (EO) is also evaluated to verify the feasibility of the EC BCI. In the experiment, two different conditions were designed which are MA that is one of the most robust mental tasks for the control task and the imagination of the English alphabet for a baseline task (**BL**), respectively. We measured hemodynamic responses using a multi-channel NIRS system while subjects performed two mental tasks with EO and EC conditions, respectively, and observed the temporal characteristics of hemodynamic responses and spatial separability of the two mental tasks (MA vs BL). The performance of classifying the two mental tasks was estimated for each of the EO and EC paradigm using two conventional linear classifiers. A post-experiment questionnaire was performed to elucidate differences between the EO and EC paradigms with respect to comprehension, difficulty, discomfort, concentration and sleepiness.

## Materials and Methods

### Subjects

Eleven right-handed healthy subjects participated in this study (five males and seven females, average age: 26.3 ± 2.7 (mean ± standard deviation)). None of them reported neurological, psychiatric or other related diseases that might affect the outcomes of the current study. This study was approved by the Ethics Committee of the Institute of Psychology and Ergonomics, Berlin Institute of Technology (approval number: SH_01_20150330) and all experiments were conducted in accordance with the declaration of Helsinki. All subjects were informed about the experimental procedure and written consent was obtained before the experiment. They were financially reimbursed after the experiment.

### Instrumentation and optode placement

**Figure 1:**
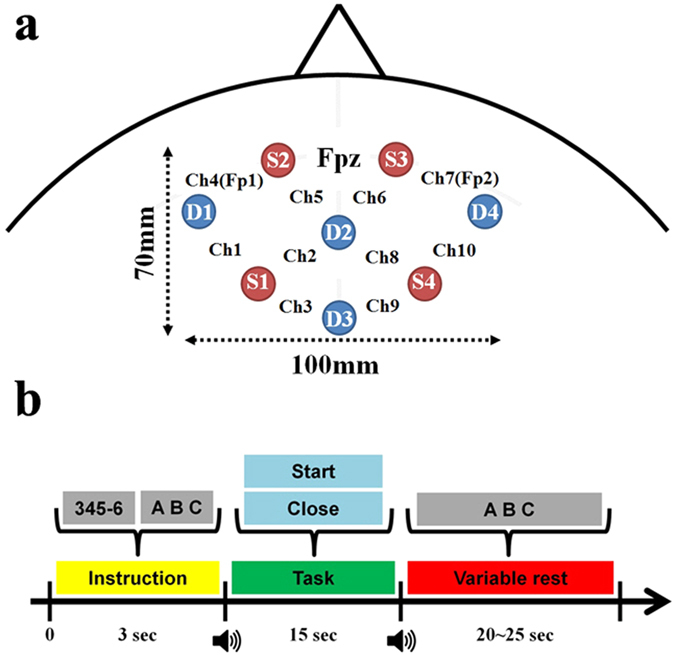
(**a**) Placement of NIRS source and detector optodes. Red (S1-4) and blue (D1-4) circles indicate the locations of source and detector optodes, respectively. NIRS channels are located between pairs of sources and detectors. Channels 1–5 and 6–10 are located on the left and right hemisphere, respectively. Channels 4 and 7 are placed on the Fp1 and Fp2 according to the international 10–10 system, respectively. (**b**) EO and EC paradigms. During an instruction, a task that the subject should perform is displayed for 3 s, e.g., 345–6 for mental arithmetic (MA), ABC for imagined vocalization of the English alphabet as a baseline task (BL). For MA, a pair of a three-digit (100–999) and one-digit (6–9) numbers are randomly displayed and varied for each trial. For the EC paradigm, the subject is asked to close his/her eyes as soon as he/she knows which task (either MA or BL) will be performed during the instruction period. The task period starts with a short beep sound. For the EO paradigm, the word ‘Start’ is displayed and the subject starts performing a given task. For the EC paradigm, ‘Close’ is displayed during the task period but the subject cannot see the word because the subject already closed the eyes before the presentation of ‘Close’. After a task period of 15 sec, a variable rest period starts with another beep sound during which the word ‘ABC’ is displayed. For the EO paradigm, the subject starts imagined vocalization of the English alphabet as controlled rest, while the subject first opens his/her eyes as soon as he/she hears the beep sound and starts imagined vocalization of English alphabet for the EC paradigm. Note that the imagined vocalization is used for both BL and controlled rest in the rest period.

NIRS data were collected by NIRSport (NIRx GmbH, Berlin, Germany) at a 15.6 Hz sampling rate. Pairs of four light sources and detectors were placed over the forehead (Fig. 1a). A customized equidistance cap was made especially for employing the forehead over the anterior prefrontal cortex. The sources and detectors were fixated in the custom-made stretchy fabric cap (EASYCAP GmbH, Herrsching, Germany). An adjacent source-detector distance was kept as 30 mm, which is a well-established setting for measuring brain hemodynamic responses^31^. An NIRS channel shown in Fig. 1a indicates the location between a pair of a source and a detector. Ch. 1–5 and ch. 6–10 were located at the left and right hemisphere of the prefrontal cortex, respectively. Ch. 4 and ch. 7 were located on the Fp1 and Fp2 positions of the international 10–10 system that is the standard attachment method for measuring EEG. No specific method was applied to further project hemodynamic responses on the cortex level, which is a common way to monitor brain hemodynamic responses in NIRS studies^5^,^7^,^18^,^20^,^32^.

### Experimental Paradigm

The subject sat on a comfortable armchair in front of a 24 inch LCD monitor and was asked not to move his/her head during the experiment to prevent motion artifact. For the EO paradigm, subjects performed MA with their eyes open, and for EC paradigm with their eyes closed. Note that the BL task was used as a baseline task during the task period and also as the controlled rest during the variable rest period for both paradigms, and it was always performed with eyes open. Six experimental runs were executed (three runs for EO and EC, respectively). The EO and EC paradigms were alternately performed across six runs for counter-balance. Each experimental run consisted of resting state without any thought (1 min), preparation to start (15 sec), 20 repetitions of a given task (10 repetitions for MA and 10 repetitions for BL for 15 sec), variable resting periods (20–25 sec) between the 20 repetitions and another resting state in the end (1 min).

During the task period, the subject performed either MA as a control task or imagined vocalization of the English alphabet as a BL. Each task was repeated ten times with pseudo-random order in each experimental run. MA comprised subtractions of a one-digit number between 6 and 9 from a three-digit number (e.g., 123). The subject was asked to perform to repetitively subtract a one-digit number from the result of previous subtraction as fast as possible (e.g., 123 − 8 = 115, 115 − 8 = 107, 107 − 8 = 99 …). For imagined vocalization of alphabet, the subject was asked to think the English alphabet from A to Z with a constant speed (1 Hz) without vocalization. This type of task is called ‘controlled rest’ in order to keep the same and constant level of light cognitive load during the resting period. This controlled rest was used because people tend to randomly think something that might disturb low loading state in a conventional resting state. Figure 1b shows a schematic diagram of the experimental paradigm.

### Data Analysis

MATLAB (R2013b; MathWorks, Natick, USA) was used for data analysis. The modified Beer-Lambert law was applied to calculate hemodynamic responses from light intensity changes. Absorption coefficient and differential pathlength factor (DPF) were used as in Fazli *et al*.^7^. To remove physiological and instrumental noise, a Chebyshev type II zero-phase band-pass filter was used (order: 6, passband: 0.01–0.2 Hz). Note that the effect of Mayer waves generally observed around 0.1 Hz was checked by applying a band-pass filter with a passband of 0.01–0.09 Hz to the NIRS data, but no significant difference was made in terms of classification accuracy and hemodynamic responses, compared to a band-pass filtering with a passband of 0.01–0.2 Hz. Thus, all the results that will be presented were obtained from the band-pass filtered NIRS data between 0.01–0.2 Hz. The number of trials was thirty for MA and BL each, which was identical for both EO and EC paradigms. Baseline correction was performed by subtracting the average value of the NIRS data measured between −2 and 0 s from each data point. We calculated the point-biserial correlation coefficient (*r-value*) to visualize the spatial distribution of separability. The *r-value* is a good measure of separability in spatial domain. The *r-value* at the time of interest is defined as^33^:


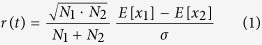


where *t* ∈ [1, 2, …, *T*], *t* is the length of the time of interest, *N*_1_ and *N*_2_ denote the total numbers of trials of class 1 and class 2, respectively. *E*[*x*_1_] and *E*[*x*_2_] are the values averaged over all trials of each class at a given time period. *σ* gives the standard deviation of all trials of both classes. For classification, various features have been used in previous NIRS-based BCI studies such as mean, slope, power, standard deviation of signal amplitude and filter coefficients^34^. Because mean and average slope of Δ[HbO] and Δ[HbR] have been most widely used^34^, we used them as features for classifiers’ input in this study. Mean and average slope of the Δ[HbO] and Δ[HbR] were calculated within a sliding time window (window size: 3 sec, step size: 1 sec, 33.3% overlap) at time period −5 to 25 s from task onset, yielding 31 window bins. The features were calculated on all the channels shown and all of them were used for classification. Feature vectors were normalized to have zero mean and unit variance.

Ten times of 5-fold cross validation were performed with two linear classification methods which are most commonly used in NIRS-based BCI studies: support vector machine with linear kernel (SVM; MATLAB statistics toolbox) and shrinkage linear discriminant analysis (shrinkage LDA, shortly LDA hereafter; the BBCI toolbox)^35^,^36^. Thirty trials for each class (i.e., MA vs BL) were randomly divided into five folds. Four folds and the remaining fold were used for training and test data set, respectively. This process was repeated ten times, leaving one different fold for testing a trained classifier in each repetition, where identical training and test samples were used for constructing two classifiers (SVM and LDA). The data measured in EO and EC states were analyzed separately. We calculated the classification accuracies for Δ[HbO] and Δ[HbR] separately, and also for a combination of Δ[HbO] and Δ[HbR] using a meta-classification method^7^. As a meta-classifier, we separately used both SVM and LDA whose weights were re-estimated within each cross-validation step to avoid a bias in the estimation of the generalization error^37^.

### Post Experiment Questionnaire

After completing the whole experiment, the subjects conducted a simple post-experiment questionnaire to determine their different impression between EO and EC states. They rated each question on a 5-point scale separately for each EO and EC state. The questionnaire included five items, as follows:

1. Comprehension: level of understanding about what they should do or should not do (1 point: no – 5 points: perfect).
2. Difficulty: level of task difficulty (1 point: very easy – 5 points: very difficult).
3. Discomfort: level of discomfort during mental task (1 point: very comfortable – 5 points: very uncomfortable).
4. Concentration: level of concentration during mental task (1 point: very low – 5 points: very high).
5. Sleepiness: level of sleepiness during the experiment (1 point: not sleepy – 5 points: very sleepy).

## Experimental Results

### Temporal Hemodynamic Response and Spatial r-value Distribution

**Figure 2:**
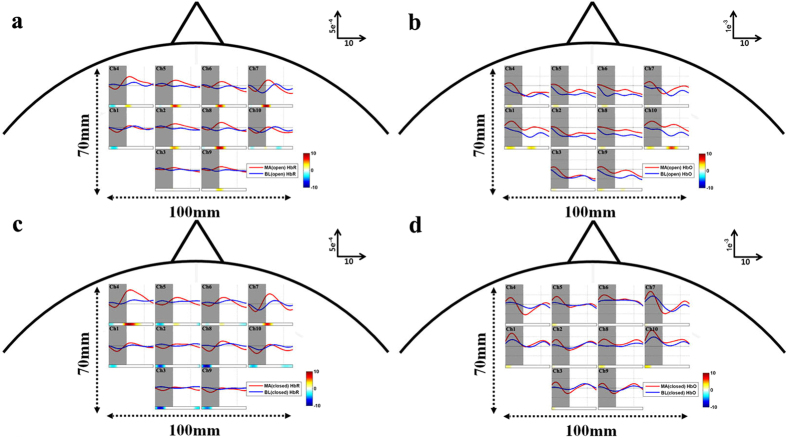
Grand-average of hemodynamic responses over whole channels for MA (red) and BL (blue): (**a**) Δ[HbR] for EO, (**b**) Δ[HbO] for EO, (**c**) Δ[HbR] for EC and (**d**) Δ[HbO] for EC paradigm. The units of x-axis and y-axis are second (s) and mmol/L. Large gray patches indicate task periods: [0 10] s. Horizontal colorbars below each plot show log(*p*). The *p-values* were corrected based on Bonferroni-correction. Vivid red and blue colors indicate significantly different time periods between MA and BL.

Figure 2a,b show the grand-average of temporal concentration changes of HbR (Δ[HbR]) and HbO (Δ[HbO]) measured with EO for MA and BL, respectively and Fig. 2c,d with EC. Horizontal colorbars below each plot show log(*p*) that were calculated at each time point. Vivid red and blue colors indicate significantly different time periods between MA and BL. Note that red and blue colors denote higher hemodynamic responses for MA than BL and BL than MA, respectively. The *p-values* calculated by the *r-values* were used for statistical tests, and Bonferroni correction was used for multiple comparisons throughout the study. Generally the largest Δ[HbR] with EO is observed while MA is performed at the frontal and center channels (red lines of ch. 2, 4–8 in Fig. 2a) and a subtle Δ[HbR] decrease is generally observed for BL (blue lines in Fig. 2a). A preceding Δ[HbO] increase by MA followed by a large Δ[HbO] decrease in EO state is observed while Δ[HbO] decreases BL in EO state (red and blue lines in Fig. 2b, respectively). Δ[HbR] increases by MA in EC state on the frontal areas; strong increase at ch. 4 and 7 and subtle increase at ch. 5 and 6 (Fig. 2c). Unlikely MA in EO state, the steep preceding Δ[HbO] increase is followed by Δ[HbO] decrease when performing MA in EC state (Fig. 2d).

**Figure 3:**
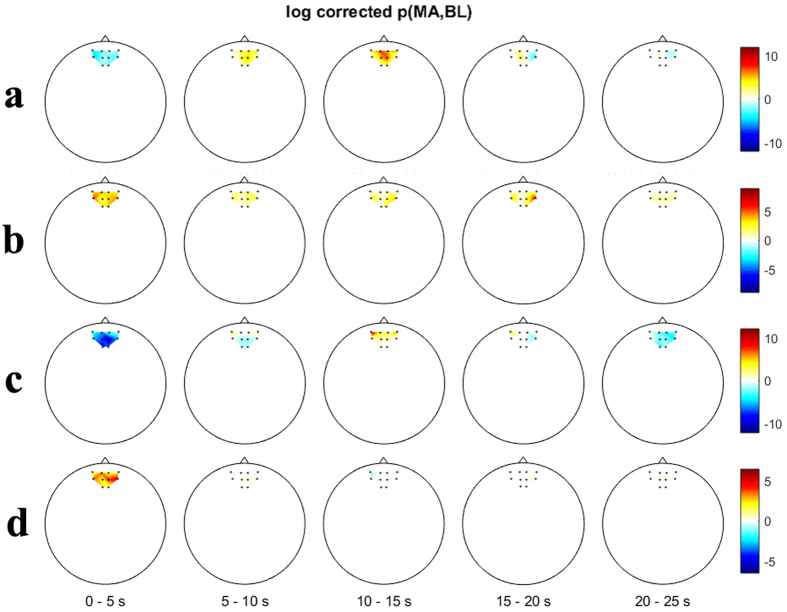
Spatial distribution of log(*p*) at given time periods of (**a**) Δ[HbR] for EO, (**b**) Δ[HbO] for EO, (**c**) Δ[HbR] for EC and (**d**) Δ[HbO] for EC. The time information below the figures indicates the time periods used for calculating log(*p*). A colorbar indicates the level of log(*p*).

In order to explore the spatial distribution of separability over the channels, Fig. 3a,b show time-dependent log(*p*) of Δ[HbR] and Δ[HbO] for EO, respectively and Fig. 3c,d for EC at time periods of interest. Note that the sign of log(*p*) at the time period 0–5 s is reversed at 5–10 s in Fig. 3a,c since the temporal Δ[HbR] induced by MA first decreased between 0–5 s and rose above the Δ[HbR] induced by BL between 5–10 s in both EC and EO conditions.

### Classification Accuracy

**Figure 4:**
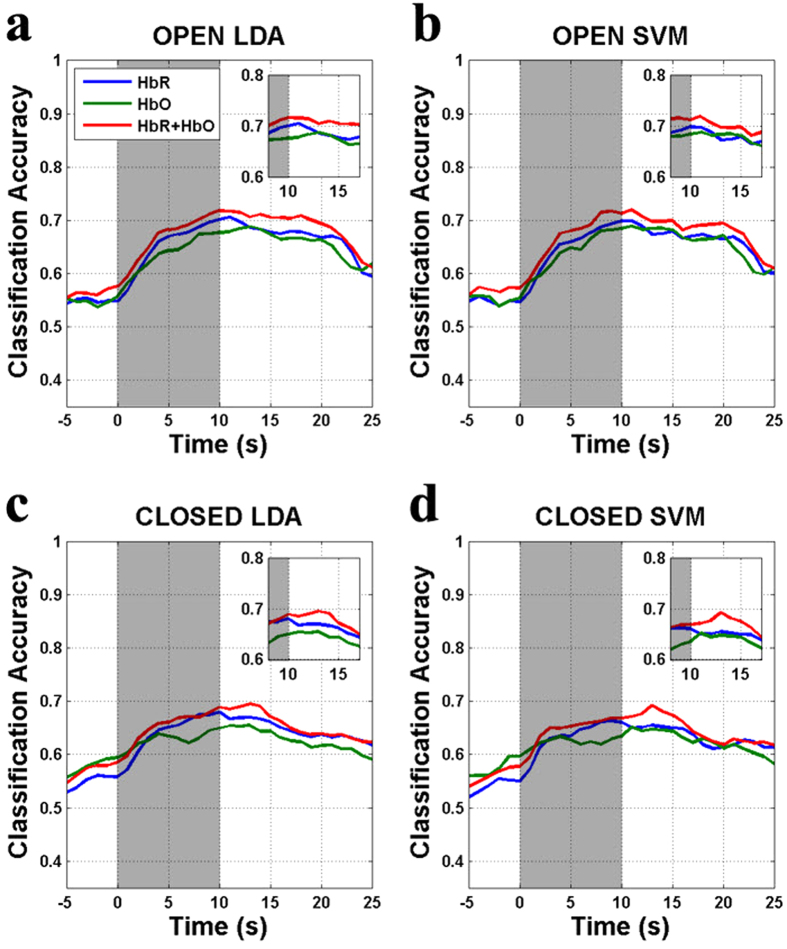
Grand-average of classification accuracies as a function of time: (**a**) SVM and (**b**) LDA and for EO and (**c**) SVM and (**d**) LDA for EC. Gray patch denotes the task period: [0 10] s. The classification accuracy is estimated using the feature vectors by sliding time window (window size: 3 s, step size: 1 s, 33.3% overlap). X-axis presents the right end of the sliding time window. Y-axis indicates the classification accuracy.

The grand-average classification accuracies are shown as a function of time in Fig. 4 and the LDA/SVM individual maximum classification accuracies with EO and EC paradigm (*maxacc*^*open*^ and *maxacc*^*closed*^, respectively) are presented in Table 1. The individual maximum classification accuracies were estimated between 5–25 s considering hemodynamic delay. In Fig. 4, the LDA/SVM average classification accuracies for HbR (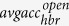), HbO (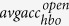) and HbR + HbO (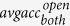) reach the maximum values at around t = 10–13 s. and LDS/SVM 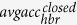, 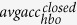 and 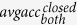 peak at around t = 9–13 s. The LDA/SVM global peak *avgacc*^*open*^are nearly the same and the LDA/SVM global peak *avgacc*^*closed*^ are also nearly same. The 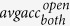 is higher than 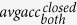 at t = 4–23 s but significant only at t = 17–18 (LDA) and 20 (SVM) s (paired t-test, *p* < 0.05). In Table 1, even though the overall classification accuracy is lower with EC than with EO, the individual *maxacc*^*closed*^ mostly exceed 70% BCI threshold for binary communication, regardless of NIRS chromophore^38^,^39^. Most of LDA *avgacc*^*open*^ and *avgacc*^*closed*^ do not differ significantly from those of SVM but the difference between LDA 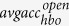 and SVM 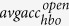 is significant (*p* = 0.0203).

**Figure 5:**
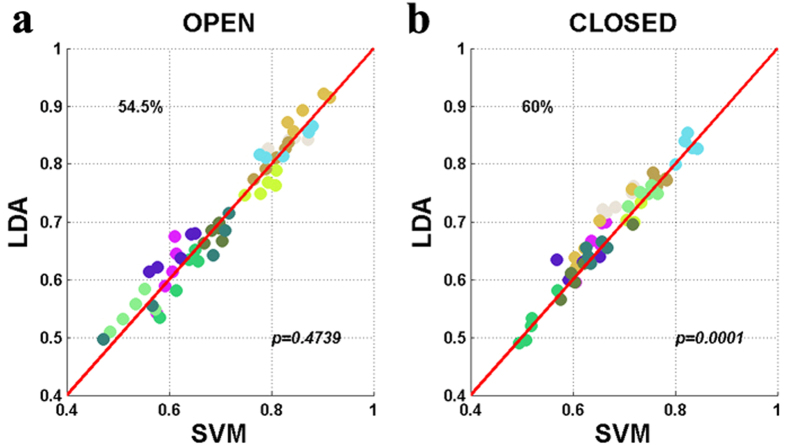
Scatter plot for comparison of classification accuracies of SVM and LDA for (**a**) EO and (**b**) EC state. The subject-dependent color is applied to circles. Each symbol indicates the classification accuracies for each subject estimated at each time point in the time period t = 5–20 s.

Figure 5a,b show the scatter plots where the respective SVM 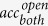 and 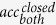, are plotted against those of LDA at a time period: [9 13] s, showing the highest classification accuracy. Circles above a red diagonal line indicate that LDA 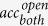 and 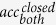
are higher than the SVM ones at the given time period. A subject-dependent color is applied to the circles. The numbers in the top-left of Fig. 5a,b indicate the ratios of higher LDA 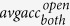 and 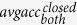, respectively. The *p-values* in Fig. 5a,b indicate statistical significance. LDA is only significantly advantageous at the given time period for EC paradigms (*p* = 0.0001).

**Figure 6:**
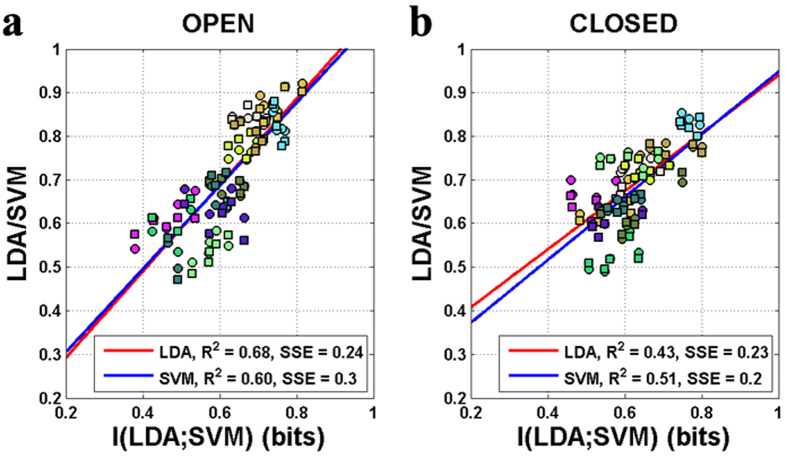
Scatter plot for comparison of mutual information and classification accuracies of SVM and LDA for (**a**) EO and (**b**) EC state. The subject-dependent color is applied to circles. Each symbol indicates the relationship between the mutual information and classification accuracy for each subject estimated at each time point in the time period t = 5–20 s.

Figure 6a,b present the relationship between mutual information of both classifier outputs and the classification accuracies of each subject at the given period: [9 13] s. Circles and squares indicate the relationship between mutual information and the LDA/SVM 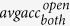 (Fig. 6a) and 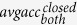 (Fig. 6b), respectively, at the given time period. Red and blue diagonal lines are linear regression lines. For both LDA and SVM, mutual information and individual classification accuracies have a similar linear correlation, in other words, each subject’s classification accuracy increases as the mutual information increases. It indicates that SVM and LDA yield similar decisions for test samples if the classification accuracy is high, while they obtain different outputs when the classification accuracy is low.

### Post Experiment Questionnaire

**Figure 7:**
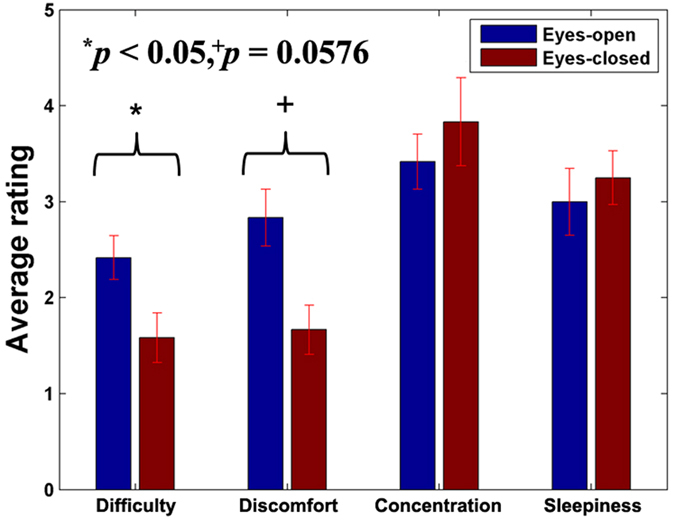
Average rating of post-experiment questionnaire on 5-point scale. Difficulty (1: very easy – 5: very difficult) and discomfort (1: very comfortable – 5: very uncomfortable) ratings are significantly and marginally higher in EO state than that in EC state. (Wilcoxson signed rank sum test, *p* < 0.05 and *p* = 0.0576, respectively).

Figure 7 shows the average rating scores regarding difficulty, comfort, concentration and sleepiness for EO and EC paradigm, respectively. The error-bar indicates the standard error of the questionnaire rating scores. Because all the subjects rated 5 points for comprehension, the comprehension score was not included. Nine out of eleven subjects reported that performing MA in EC state was easier than that in MA in EO state. In line with this, difficulty rating of EO is significantly higher than EC (*p* < 0.05). Discomfort rating for EC results is marginally low in terms of statistical significance (*p* = 0.0576). Concentration and sleepiness rating for EC state were slightly higher than that for EO state but not significant.

## Discussion

We exploited MA and BL as the intentional mental task and controlled resting task with very light work load instead of normal resting state because a pure resting state (resting state without any thought) is not possible to achieve in practice. Given that MA and BL evoked larger and smaller APFC activation, we discriminated MA-related activations from BL-related ones that were normally weaker in both EO and EC states. As a result, acceptable classification accuracy was attained for both EO and EC conditions (>70%), demonstrating the potential feasibility of the EC NIRS-based BCIs.

### Necessity of EC NIRS-based BCI

For patients severely suffering from neuromuscular disorders it is difficult to keep their eyes open consistently. Previous studies introduced non-visual BCIs such as auditory and tactile BCI for patients with ocular dysfunction^40^,^41^,^42^,^43^,^44^,^45^,^46^,^47^,^48^. Recently, the EC BCI paradigm was newly proposed based on the SSVEP^28^ or event-related potentials (ERP)^29^. However, it is not suitable to use fast task-relevant response for NIRS-based BCI since it cannot be clearly detected by means of NIRS because of its inherent hemodynamic delay^49^. In this study, therefore, we established the EC BCI paradigm using the PFC activations evoked by MA under EC state. Because users do not have to keep their eyes open and blinking while using the EC BCI, it is convenient and comfortable for oculomotor impaired patients, compared to traditional EO BCIs. Since it was revealed that EC BCI could present a competitive performance in comparison with a conventional EO BCI (HbR + HbO with EO and EC: 77.0 ± 9.2% and 75.6 ± 7.3% (LDA), respectively. *p* = 0.5698) the proposed EC BCI paradigm could be regarded as a successful new BCI paradigm.

### Spatial Pattern and Classification

Pfurtscheller *et al*.^17^ reported Δ[HbR] increase and Δ[HbO] decrease evoked by simple calculation at APFC. We also speculated about the presence of a similar pattern over APFC during MA and weak (or no) activation during BL. With the help of visual inspection of the grand-average temporal hemodynamic responses (Fig. 2), the anterior channels (ch. 4–7) showed the expected pattern of hemodynamic responses. In light of classification, because the absolute *r-value* of grand-average Δ[HbR] is larger than that of grand-average Δ[HbO] around t = 10 s where the maximum classification performance is obtained, Δ[HbR] can be more classifiable than Δ[HbO] (Fig. 3). Nevertheless, *acc*_*hbr*_ and *acc*_*hbo*_ do not significantly differ; rather *acc*_*hbo*_ scores higher in some cases (Table 1). Hence, HbR can be more suitable for EC BCI paradigm to obtain the stable and good performance.

### EO vs EC Paradigm

The mean classification performance obtained in EO state was generally higher than that obtained in EC state. The difference between *acc*^*open*^ and *acc*^*closed*^ may originate from the different task difficulty. Subjects reported that they felt performing MA in EC state was easier and more comfortable than in EO state. By closing their eyes, they could easily reject the ambient visual stimuli; therefore, they could focus more on performing the given task. Herff *et al*.^50^ reported the more difficult a task was, the higher classification accuracy was achievable in the experiment regarding PFC mental task because higher brain activity is produced. Since difficulty and discomfort rating was significantly and marginally higher in EO state than in EC state, respectively, we infer that the level of difficulty and discomfort results in the difference of *acc*^*open*^ and *acc*^*closed*^. Nevertheless, since the difference between *acc*^*open*^ and *acc*^*closed*^was not significant over most of the time periods, APFC activation with EC is expected to be useful for developing practical NIRS-based EC BCIs.

### Study Limitation and Future Work

In principle, subjects had to keep their eyes closed during the experiment for EC paradigm. However, in this study, subjects were asked to open and close their eyes repeatedly to maintain their concentration and to prevent them from falling asleep for EC paradigm. We did not identify the effect of opening and closing their eyes on the BCI performance. On the other hand, NIRS has inferior temporal responsiveness due to inherent hemodynamic delay. It needs a longer stimulus and rest period than that of EEG-based BCI. Also, it usually takes a long time to detect user’s intention which could result in practical limitations. In the recent years, EEG-NIRS hybrid BCI research is emerging^51^,^52^. Taking advantage of the merit of the hybrid BCI, the potential drawback of NIRS-based BCI systems can be compensated in terms of temporal responsiveness and accuracy^7^,^53^.

Recently, many BCI studies are focusing on on-line BCI and on-line implementation is essential to confirm the practical usability of BCI paradigm^29^,^54^,^55^. We evaluated the feasibility of NIRS-based EC BCI as an alternative of conventional BCI paradigms and performed off-line analyses. In addition, pseudo on-line classification results are provided as supplementary information. A full on-line NIRS-based EC BCI deserves a full own study as other important scientific and technical questions need to be analyzed in order to understand also possible on-line limitations of the novel paradigm.

## Conclusion

In this study, we first investigated the feasibility of NIRS-based EC BCI using APFC activation. From the statistical analysis result of the post-experiment questionnaire, we concluded that lower difficulty would result in lower classification accuracy for EC. Nevertheless, the proposed EC BCI achieved the comparable classification accuracy to EO BCI. It could be utilized as a new BCI paradigm for patients who have difficulties to their eyes during an experiment or completely locked-in patients. It is easier to concentrate on performing tasks in the EC state and a more comfortable experimental condition for many subjects.

## Additional Information

**How to cite this article**: Shin, J. *et al*. Near-infrared spectroscopy (NIRS)-based eyes-closed brain-computer interface (BCI) using prefrontal cortex activation due to mental arithmetic. *Sci. Rep.* **6**, 36203; doi: 10.1038/srep36203 (2016).

**Publisher’s note:** Springer Nature remains neutral with regard to jurisdictional claims in published maps and institutional affiliations.

## Figures and Tables

**Table 1:** 

(a) Eyes-open						
subject	LDA			SVM		
	HbR	HbO	HbR + HbO	HbR	HbO	HbR + HbO
VP001	62.0	69.7	68.8	62.0	69.8	65.5
VP002	**86.8**	**87.7**	**88.8**	**88.7**	**90.5**	**91.5**
VP003	**70.8**	62.8	**70.0**	68.5	68.2	**70.8**
VP004	**93.3**	**84.3**	**92.2**	**91.5**	**86.2**	**91.3**
VP005	**76.5**	**72.7**	**78.8**	**81.0**	**76.2**	**81.0**
VP006	68.8	**71.2**	69.2	67.3	**72.5**	69.0
VP007	**84.0**	**78.5**	**83.7**	**82.2**	**80.5**	**85.0**
VP008	59.0	67.3	64.2	59.0	65.0	61.8
VP009	**85.2**	**86.3**	**87.8**	**86.3**	**86.5**	**88.0**
VP010	65.2	**72.2**	**71.7**	66.8	**73.7**	**72.8**
VP011	**70.5**	67.0	**71.5**	**70.5**	68.7	**71.7**
**Mean**	74.7	74.5	77.0	74.9	76.2	77.1
**Std**	10.7	8.1	9.2	10.8	8.2	10.1
**(b) Eyes-closed**						
**subject**	**LDA**			**SVM**		
	**HbR**	**HbO**	**HbR + HbO**	**HbR**	**HbO**	**HbR + HbO**
VP001	64.7	**70.3**	**70.0**	63.7	69.8	66.3
VP002	**88.2**	**83.8**	**86.3**	**83.2**	**80.0**	**83.2**
VP003	61.2	59.8	62.2	64.7	59.8	63.2
VP004	**74.0**	**76.5**	**78.5**	**73.3**	**75.0**	**75.7**
VP005	**75.5**	64.5	**74.8**	**74.5**	65.0	**74.8**
VP006	68.5	66.5	69.8	66.3	68.7	66.3
VP007	**80.0**	**73.5**	**79.2**	**79.7**	**72.8**	**80.0**
VP008	**80.3**	**80.3**	**80.2**	**80.2**	**79.3**	**80.5**
VP009	**74.5**	**86.8**	**85.3**	**76.3**	**84.0**	**84.3**
VP010	**77.5**	**77.0**	**78.7**	**76.8**	**82.2**	**78.7**
VP011	68.0	66.7	66.5	64.7	67.3	66.7
**Mean**	73.8	73.3	75.6	73.0	73.1	74.5
**Std**	7.4	8.1	7.3	6.7	7.4	7.3
